# Persistent Sick Sinus Syndrome in Scrub Typhus

**DOI:** 10.4269/ajtmh.16-0909

**Published:** 2017-05-03

**Authors:** Lae-Young Jung, Ji-Young Yoon, Soo-Kyeong Song, Chang-Seop Lee

**Affiliations:** 1 Department of Internal Medicine, Chonbuk National University, Jeonju, Republic of Korea; 2 Biomedical Research Institute of Chonbuk National University Hospital, Jeonju, Republic of Korea

**Figure 1.**
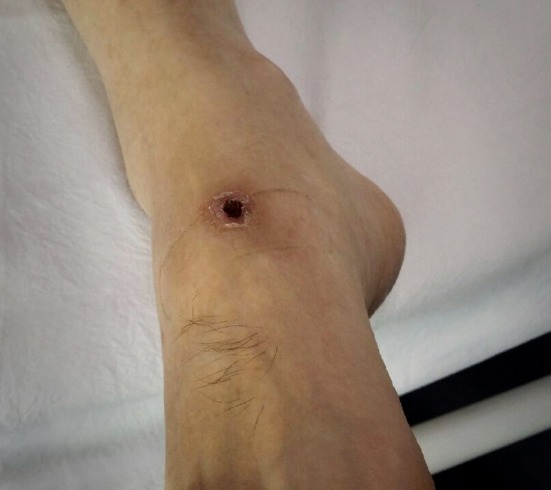
Eschar present in the ankle region.

**Figure 2.**
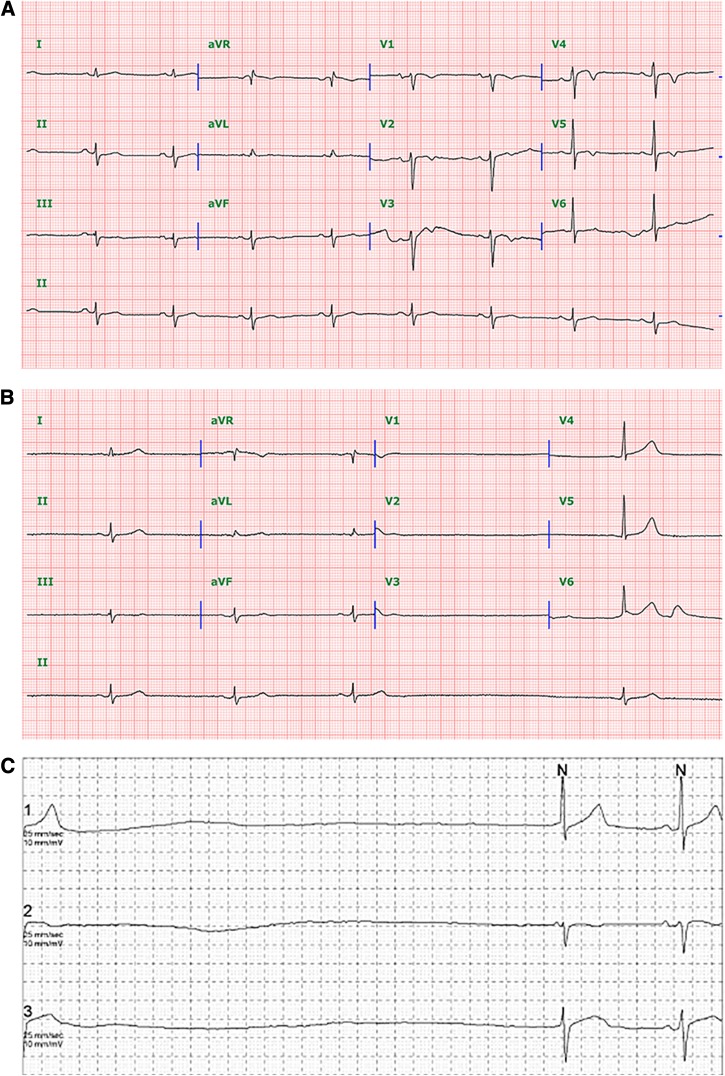
(**A**) The initial electrocardiogram (ECG) showed normal sinus rhythm. Heart rhythm controlled by sinus node at a rate of 56 beats/minute and each P wave followed by QRS. (**B**) After a few days, ECG showed marked sinus bradycardia and sinus arrest with junctional escape rhythm. Three sinus beats are followed by an interval with no P waves. A junctional escape rhythm then emerges with QRS complex the same as in sinus rhythms. (**C**) ECG telemetry showed marked bradycardia with sinus arrest. After initial T wave, 5.84 seconds sinus pause is followed by a junctional escape rhythm.

A 58-year-old man without comorbidity presented with high fever, chills, and myalgia. Examination showed right inguinal lymphadenopathy; an eschar was present in the ankle region (Figure 1

). His blood pressure was 100/60 mmHg, heart rate was 70 beats/minute, and the electrocardiogram (ECG) showed normal sinus rhythm (Figure 2A

). The platelet count was 74,000/μL, total bilirubin was 1.3 mg/dL, aspartate aminotransferase was 105 IU/L, and alanine aminotransferase was 84 IU/L. Chest radiograph was normal; abdominal computed tomography showed mild hepatosplenomegaly with a small amount of ascites. Blood and urine cultures were negative. IgG immunofluorescence assay titer for *Orientia tsutsugamushi* was 1:5,120. Intravenous azithromycin was given for 3 days followed by oral doxycycline for 4 more days. Ten days later dizziness and fatigue occurred. On examination, the heart rate was 45 beats/minute and the ECG showed marked sinus bradycardia and sinus arrest with junctional escape rhythms (Figure 2B). Levels of cardiac enzymes were within normal limits (troponin I: 0.100 ng/mL [normal < 0.16 ng/mL]; creatine kinase-MB: 1.11 ng/mL [normal < 0.494 ng/mL]; myoglobin: 21.00 ng/mL (normal < 0.72 ng/mL]). Echocardiography showed normal cardiac function. ECG monitoring showed that dizziness corresponded to bradycardia, with marked bradycardia due to recurrent sinus arrests (maximal beat to beat interval [R-R interval] was 7.36 seconds) and an average heart rate of 32/minute (Figure 2C). Sick sinus syndrome was diagnosed, leading to placement of a permanent pacemaker, which led to symptomatic improvement. Arrhythmia has been reported as a complication of scrub typhus.^1^,^2^ Relative bradycardia is known to occur in scrub typhus cases.^3^ Most occurrences of bradycardia have been known to be transient and reversible events. This report describes the first observation of irreversible bradycardia associated with scrub typhus infection leading to placement of a permanent pacemaker.
